# Supplementary material for: Prediction of Neonatal Respiratory Morbidity Assessed by Quantitative Ultrasound Lung Texture Analysis in Twin Pregnancies
Source: J Clin Med. 2022 Aug 20;11(16):4895. doi: 10.3390/jcm11164895 (PMC9409975; doi:10.3390/jcm11164895)
Supplement: Supplementary file 1 [file jcm-11-04895-s001.zip › jcm-1836095-supplementary.pdf]

Supplementary Table S1

Summary of excluded cases due to administration of antenatal steroids after image acquisition and before delivery.

Case	LMP	Date Scan	GA Scan	Date Steroids	Date delivery	GA delivery	QFLM risk	RDS	TTN	Type result
1	31/5/17	19/1/18	33.4	19-20/1/18	21/1/18	33.6	Low	No	Yes	False negative
1	31/5/17	19/1/18	33.4	19-20/1/18	21/1/18	33.6	Low	No	No	True negative
2	1/7/18	1/3/19	34.5	1-2/3/19	3/3/19	35.0	Low	No	No	True negative
3	12/5/18	9/1/19	34.4	9-10/1/19	10/1/19	34.5	Low	No	No	True negative
3	12/5/18	9/1/19	34.4	9-10/1/19	10/1/19	34.5	Low	Yes	No	False negative
4	25/9/17	22/5/18	33.5	22-23/5/18	24/5/18	34.0	Low	No	Yes	False negative
4	25/9/17	22/5/18	33.5	22-23/5/18	24/5/18	34.0	Low	No	No	True negative
5	16/12/18	26/7/19	31.5	26-27/7/19	29/7/19	32.1	High	No	Yes	True positive
5	16/12/18	26/7/19	31.5	26-27/7/19	29/7/19	32.1	High	No	Yes	True positive
6	12/6/18	30/10/18	32.3	16-17/10/18	3/11/18	33.0	Low	No	No	True negative
6	12/6/18	30/10/18	32.3	16-17/10/18	3/11/18	33.0	Low	No	No	True negative
7	8/1/19	3/9/19	34.0	5/9/19	5/9/19	34.2	Low	No	No	True negative
7	8/1/19	3/9/19	34.0	5/9/19	5/9/19	34.2	Low	No	No	True negative

LMP: last menstrual period. GA: gestational age. QFLM: quantus fetal lung maturity test. RDS: respiratory distress syndrome. TTN: transient tachypnea of the newborn

Supplementary Table S2

Summary of performance of quantusFLM® in general population and in twin pregnancies to predict neonatal respiratory morbidity

	Accuracy	Sensitivity	Specificity	PPV	NPV	F1-Score
*General	91.5	71.0	94.7	67.9	95.4	69.4
Twin	89.2	42.9	95.9	60.0	92.1	50.0

*Burgos-Artizzu XP, et al. *Evaluation of an improved tool for non-invasive prediction of neonatal respiratory morbidity based on fully automated fetal lung ultrasound analysis. Sci Rep 2019*.

Supplementary Table S3

Pre-test risks and probabilities, positives and negatives likelihood ratios and a post-test probabilities and risks of the neonatal respiratory morbidity in twin pregnancies

Edad	A priori risk	A priori probability	LR +	A posteriori probability (LR+)	LR -	A posteriori probability (LR-)	A posteriori risk (LR+)	a posteriori risk (LR-)
<30	87,50%	7,00	22,30	156,10	0,48	3,36	99,4%	77,06%
30-31	73,50%	2,77	22,30	61,85	0,48	1,33	98,4%	57,11%
32-33	41,20%	0,70	22,30	15,63	0,48	0,34	94,0%	25,17%
34-34.6	19,80%	0,25	1,20	0,30	0,90	0,22	22,9%	18,18%
35-35.6	8,90%	0,10	1,20	0,12	0,90	0,09	10,5%	8,08%
36-36.6	4,50%	0,05	1,20	0,06	0,90	0,04	5,4%	4,07%

+/- LR: positive/negative likelihood ratios.
